# MVSF-AB: accurate antibody–antigen binding affinity prediction via multi-view sequence feature learning

**DOI:** 10.1093/bioinformatics/btae579

**Published:** 2024-10-03

**Authors:** Minghui Li, Yao Shi, Shengqing Hu, Shengshan Hu, Peijin Guo, Wei Wan, Leo Yu Zhang, Shirui Pan, Jizhou Li, Lichao Sun, Xiaoli Lan

**Affiliations:** School of Software Engineering, Huazhong University of Science and Technology, Wuhan 430000, China; School of Software Engineering, Huazhong University of Science and Technology, Wuhan 430000, China; Department of Nuclear Medicine, Union Hospital, Tongji Medical College, Huazhong University of Science and Technology, Wuhan 430000, China; School of Cyber Science and Engineering, Huazhong University of Science and Technology, Wuhan 430000, China; School of Cyber Science and Engineering, Huazhong University of Science and Technology, Wuhan 430000, China; School of Cyber Science and Engineering, Huazhong University of Science and Technology, Wuhan 430000, China; School of Information and Communication Technology, Griffith University, Queensland 4222, Australia; School of Information and Communication Technology, Griffith University, Queensland 4222, Australia; School of Data Science, City University of Hong Kong, Hong Kong 999077, China; Department of Computer Science and Engineering, Lehigh University, Bethlehem, PA 18018, United States; Department of Nuclear Medicine, Union Hospital, Tongji Medical College, Huazhong University of Science and Technology, Wuhan 430000, China

## Abstract

**Motivation:**

Predicting the binding affinity between antigens and antibodies accurately is crucial for assessing therapeutic antibody effectiveness and enhancing antibody engineering and vaccine design. Traditional machine learning methods have been widely used for this purpose, relying on interfacial amino acids’ structural information. Nevertheless, due to technological limitations and high costs of acquiring structural data, the structures of most antigens and antibodies are unknown, and sequence-based methods have gained attention. Existing sequence-based approaches designed for protein-protein affinity prediction exhibit a significant drop in performance when applied directly to antibody–antigen affinity prediction due to imbalanced training data and lacking design in the model framework specifically for antibody–antigen, hindering the learning of key features of antibodies and antigens. Therefore, we propose MVSF-AB, a Multi-View Sequence Feature learning for accurate Antibody–antigen Binding affinity prediction.

**Results:**

MVSF-AB designs a multi-view method that fuses semantic features and residue features to fully utilize the sequence information of antibody–antigen and predicts the binding affinity. Experimental results demonstrate that MVSF-AB outperforms existing approaches in predicting unobserved natural antibody–antigen affinity and maintains its effectiveness when faced with mutant strains of antibodies.

**Availability and implementation:**

Datasets we used and source code are available on our public GitHub repository https://github.com/TAI-Medical-Lab/MVSF-AB.

## 1 Introduction

Antibodies play an indispensable role in disease detection, prevention, and treatment by identifying, attaching to, and counteracting invading pathogens in the immune system. The interaction between antibodies and antigens is a highly specific protein interaction, and the binding affinity between them measures the strength and effectiveness of this interaction (Milenic *et al.* 2004, Lu *et al.* 2020). Accurate quantification of antibody–antigen binding affinity is crucial for understanding disease pathogenesis and advancing diagnostic and therapeutic approaches (Ai and Advani 2015). However, traditional techniques used to assess antibody–antigen affinity, such as immunoprecipitation, ELISA, and epitope mapping (Heinrich *et al.* 2010), require significant time, resources, and harsh experimental conditions.

In the face of these challenges, artificial intelligence (AI) technology has shown remarkable potential. It can analyze vast biological datasets and make effective predictions. Thanks to machine learning and deep learning algorithms, antibody–antigen interactions and correlations can be analyzed with increased speed and comprehensiveness, leading to more accurate predictions of their affinity (Rath *et al.* 1988, Choi 2022).

Predicting the affinity of antibody–antigen binding can use the structural information or sequence information of the antibody–antigen. Existing schemes tailored for antigen–antibody affinity prediction are all based on structural information. CSM-AB (Myung *et al.* 2022) predicted antibody–antigen affinity by utilizing graph-based signatures to characterize the interfacial network of antibody–antigen interactions. AREA-AFFINITY (Yang *et al.* 2023) employed contact-based and area-based descriptors to predict antibody–antigen affinity. However, the determination of protein structures is hindered by technical limitations and substantial costs (Boutet *et al.* 2012, Yip *et al.* 2020), resulting in a scarcity of data encompassing accurate structural information. Hence, for antigen–antibody samples lacking structural information, these approaches cannot be used to predict the affinity.

Due to the vast amount of sequence data generated by high-throughput sequencing (Bill *et al.* 2011), numerous studies have been dedicated to predicting protein-protein binding affinity by analyzing the sequence feature. The first work on predicting protein-protein binding affinity using sequence feature analysis was proposed by Yugandhar and Gromiha (2014). They utilized the physicochemical properties of each amino acid as features and made several attempts to identify combinations of features that exhibited the highest correlation with affinity. Abbasi *et al.* (2020) introduced ISLAND, a method that predicts binding affinity by integrating various representations of sequence characteristics with various regression models. Chen *et al.* (2019) encoded pairs of protein sequences by employing a residual recurrent convolutional neural network (RCNN) with a Siamese architecture, capturing both local features and contextual information, and feeding the encoded sequences into a multilayer perceptron (MLP). Unsal *et al.* (2022) evaluated the performance of various protein pretrained models in characterizing sequence features and different regression models in predicting binding affinity. The pretrained model ProtALBERT (Lu *et al.* 2022), which is a BERT-based language model, demonstrates the best performance. This finding highlighted the effectiveness of the language model in extracting sequence information. However, the aforementioned sequence-based affinity prediction studies are primarily focused on the general protein-protein binding affinity and struggle to capture specific antigen–antibody interactions owing to imbalanced training data and a lack of targeted model design. Consequently, there is an imperative need to develop tailored models specifically for protein classes such as antigen–antibody.

In this work, we propose Multi-View Sequence Feature learning for accurate Antibody–antigen Binding (MVSF-AB), a novel framework specifically designed for predicting the affinity of antigen–antibody that fuses multi-view sequence features, including semantic features and residue features. On the one hand, semantic features of antibody and antigen sequences are extracted using a pretrained language model proteinBERT, followed by a convolutional neural network (CNN) to learn global correlations between the features of antibody and antigen sequences. On the other hand, residue feature matrices are constructed for each antigen–antibody complex, and a MLP is employed to learn key binding sites from the residue feature of each position. Experimental results demonstrate that MVSF-AB outperforms existing methods in accurately predicting the affinity of both natural and mutated antigen–antibody pairs. This also highlights the necessity of customizing affinity prediction models according to the characteristics of different research areas in biology.

## 2 Materials and methods

### 2.1 Datasets

At present, there is a shortage of antibody–antigen datasets offering affinity information. Table 1 enumerates some datasets that are useful for investigating the binding affinity between antibodies and antigens. Notably, “available entries” represents the number of protein antibodies in the dataset, while “total entries” represents the number of all entries, including protein antibodies, non-antibody proteins, and other types of antibodies. Only protein antibodies in the dataset are utilized in our scheme; non-antibody proteins and other types of antibodies have been removed.

**Table 1.** Overview of publicly available antibody–antigen datasets.

Dataset	Type	Total entries	Available entries
Kastrictis’ benchmark (Kastritis *et al.* 2011)	Natural	144	19
Kastrictis’ benchmark 2.0 (Vreven *et al.* 2015)	Natural	179	33
SAbDab (Dunbar *et al.* 2014)	Natural	739	579
AB-Bind (Sirin *et al.* 2016)	Mutant	1089	1089
SKEMPI (Moal and Fernández-Recio 2012)	Mutant	3047	234
SKEMPI 2.0 (Jankauskaite *et al.* 2019)	Mutant	7085	387
An expanded benchmark (Guest *et al.* 2021)	Natural	42	38

These datasets can be categorized into two main types: *natural* antibody–antigen affinity datasets and *mutated* antibody–antigen affinity datasets. Since SAbDab (Dunbar *et al.* 2014) is sourced from Kastrictis’ affinity benchmarks (Kastritis *et al.* 2011, Vreven *et al.* 2015), and SKEMPI 2.0 (Jankauskaite *et al.* 2019) is an extension of SKEMPI (Moal and Fernández-Recio 2012), we decided to use SAbDab as the dataset for predicting natural antibody–antigen affinity and opted for AB-Bind and SKEMPI 2.0 to predict the affinity of mutant antibody–antigen pairs. Furthermore, we utilized an expanded benchmark (Guest *et al.* 2021) as the test dataset to evaluate the predictive capability of the proposed method on unseen natural antibody–antigen binding affinity. We compared the distribution and sequence similarity between the benchmark and the training set SAbDab using the CD-HIT (Cluster dataset at High Identity with Tolerance) method (Li and Godzik 2006), a widely used bioinformatics tool for clustering and comparing protein or nucleotide sequences. As displayed in Supplementary Fig. S2, we can see that the benchmark and the training set are independent and identically distributed.

We selected SAbDab, an online resource that provides a comprehensive collection of publicly available antibody structures, as our primary dataset. The dataset includes various annotations such as experimental information, accurate heavy and light chain pairings, antigen details, and antibody–antigen binding affinity information. Note that the SAbDab exclusively contains data on natural antibodies. In contrast, the AB-Bind dataset provides information regarding binding affinity changes resulting from mutations. To study the impact of mutations, we screened a total of 1089 data entries from AB-Bind. Furthermore, we also screened 387 antibody data entries from the SKEMPI 2.0 dataset, which captures affinity changes after site mutations in protein-protein complexes.

We selected △G as the parameter to quantify the affinity. △G denotes the change in free energy associated with the binding of an antibody to its corresponding antigen. It indicates the energy variation within the system during the binding process, reflecting the energy released or absorbed by the antibody–antigen interaction (Kastritis *et al.* 2011). A more negative △G value indicates a more stable binding process and a stronger affinity between the antibody and antigen.

### 2.2 Overview of framework

**Figure 1.**
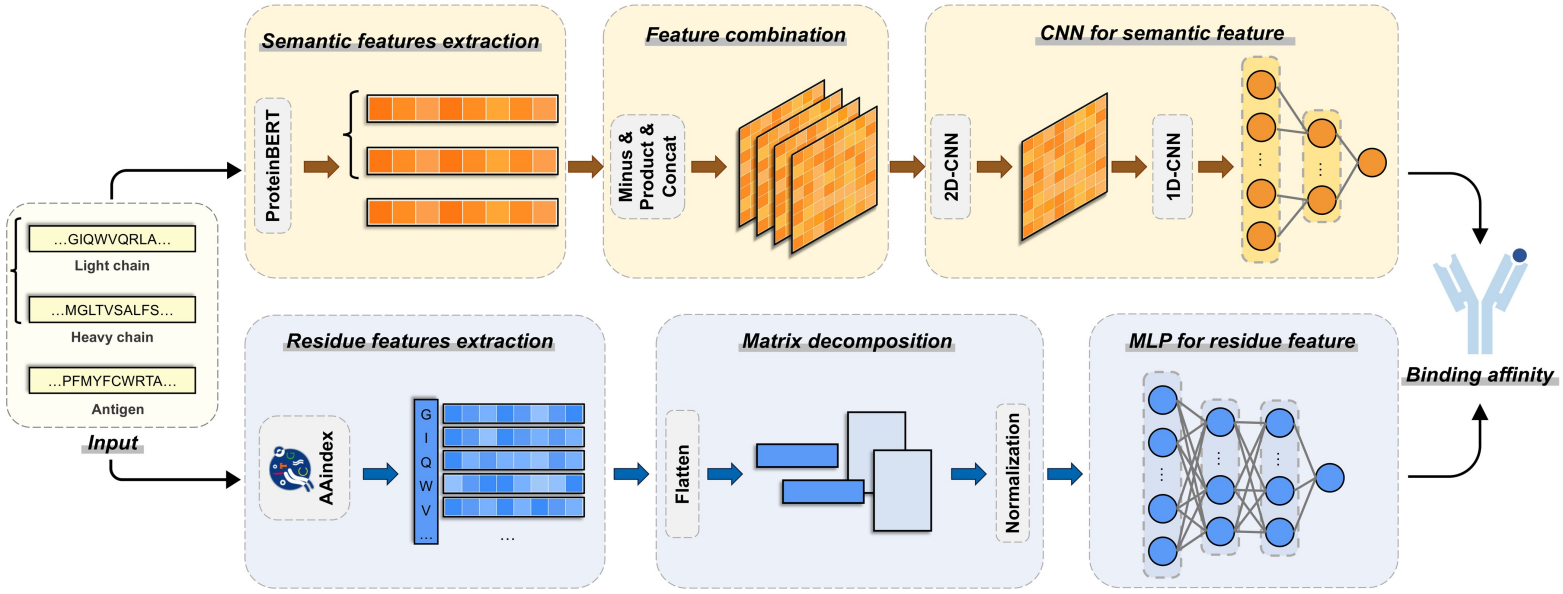
The overview of MVSF-AB. The sequences are initially processed by the embedding layer using the pretrained proteinBERT model, which generates embeddings for each sequence. These embeddings are then passed to the feature combination block to obtain the features of the antibody–antigen complexes. Subsequently, the sequence features are fed into the CNN block for compression and dimensionality reduction. Simultaneously, a feature matrix for each complex is generated by the physicochemical properties of amino acids from the AAindex dataset. This feature matrix is matrix-decomposed and subsequently fed into the MLP. Ultimately, the outputs of the CNN and the MLP are weighted and averaged to obtain the final binding affinity prediction.

MVSF-AB has developed distinct modules for sequence features and residue features, as illustrated in Fig. 1. We first extracted the sequence features of antibodies and antigens using a pretraining proteinBERT model (Brandes *et al.* 2022). Subsequently, these features are combined and input into a CNN (Zhang *et al.* 2020). We then extracted the residue feature from AAindex (Kawashima and Kanehisa 2000, Kawashima *et al.* 2007) and generated a feature matrix. Later on, we utilized a matrix decomposition technique to integrate antigen features with those of the light and heavy chains of antibodies. The resulting tensor is then utilized as input for the prediction task in the MLP (Desai and Shah 2021). Finally, the prediction outputs from both the CNN and the MLP are fused (Zhao and Zhou 2021) to derive the final predicted affinity.

### 2.3 Data preprocessing

Taking into account the distinct roles of light and heavy chains in the antibody–antigen binding process, we treated them separately. We divided each antibody–antigen complex sample into three chains, including antibody light chain, antibody heavy chain, and antigen chain. The sample *X* is defined as:

(1) X=(L,H,A,y)

where *L*, *H*, *A*, and y∈R are antibody light chain, heavy chain sequences, antigen sequences, and annotated binding affinity values, respectively. Sequence data was stored in a standardized FASTA format file and subsequently fed into the embedding layer for further processing.

For datasets containing mutation information, we modified the natural antibody sequences according to the guidelines provided to obtain the sequences of the mutants, which were then divided into three chains.

### 2.4 Semantic feature extraction

A pretrained proteinBERT model (Brandes *et al.* 2022) specifically designed for protein sequences was utilized to encode both the light and heavy chains of the antibodies, as well as the antigens, separately. Notably, the encoding length of the model was periodically switched as sequences were processed in batches. This flexibility enables the model to effectively handle sequences of varying lengths. During pretraining, we put the light chain, heavy chain, and antigen sequences from each antibody–antigen complex into separate embedding layers. Each input of amino acid sequence was converted into a comprehensive 768-dimensional vector representation that encapsulates both local residue-level details and global sequence-level information.

(2) $$L_{s}=\text{Embed}\left(L\right),H_{s}\;=\;\text{Embed}\left(H\right),A_{s}\;=\;\text{Embed}\left(A\right)$$

where $L_{s}$, $H_{s}$, and $A_{s}$ are all 768-dimensional vectors.

### 2.5 Feature combination

The antibody light chain and heavy sequences are combined with antigen sequences, respectively, which were incorporated as distinct channels into the CNN block. This approach enables the model to effectively capture the intricate relationship between each antibody sequence and antigen sequence in one complex, thereby enhancing the CNN model accuracy.

The embedding features of antibody and antigen sequences $L_{s}$, $H_{s}$, and $A_{s}$ were elevated in separate dimensions for subsequent tensor computation. The interactions are calculated by:

(3) $$D_{L}=\left|L_{s}⊖A_{s}\right|,P_{L}=L_{s}⊙A_{s}$$

where ⊖ denotes element-wise subtraction and ⊙ denotes Hadamard product. We performed the same procedure on the heavy chain and concatenated these feature matrixes to obtain the combined features of the whole complex and used layer normalization operation to normalize the feature tensor.

(4) $$C=\left[D_{L},P_{L},D_{H},P_{H}\right]$$

where $C\in R^{4\times 768\times 768}$ denotes the combined feature tensor.

### 2.6 CNN for sequence feature

Then *C* from each batch of samples were fed into the 2D CNNs to extract the feature $B\in R^{768\times 768}$ of the antibody (Roska and Chua 1993, Simonyan and Zisserman 2014).

(5) B=ReLU(Conv2d(C))

We then used 1D CNNs to compress the antibody representation into a 768-dimensional vector. Max pooling was then performed on this condensed vector to reduce the dimensionality of features, thereby retaining the most informative signals and mitigating the risk of overfitting. Finally, the extracted features were passed through a fully connected layer to learn the relationship between the antibody representation and binding affinity values (Sainath *et al.* 2015).

(6) $$A=\text{MaxPool}\left(\text{ReLU}\left(\text{Conv}1d\left(B\right)\right)\right),{\hat{\text{y}}}_{s}=\text{AW}+b$$

where *A* is a 384-dimensional vector and ${\hat{y}}_{s}\in R$.

### 2.7 Residue features extraction

AAindex is a dataset compiling numerical indices representing the physicochemical and biochemical properties of individual amino acids and amino acid pairs. We utilized the AAindex1 subset to characterize each residue in the antibody and antigen sequences. We utilized principal component analysis (PCA) to condense the 544 physicochemical indices in AAindex1 into 20 dimensions to improve computational efficiency. In addition, Supplementary Fig. S5 analyzed the importance of the top 20 features using the SHAP method.

We standardized the lengths of all light and heavy chains, as well as antigen chains, to the same length, *m*. Taking the antibody light chain sequence as an example, the same process applies to heavy and antigen chains. During the training phase, we set *m* as the length of the longest light chain for all the light chains. Then, to standardize the length, the light chains that are shorter than *m* are zero-padded. In the testing phase, to match the model’s parameters, the length of the antibody light chain is truncated to *m* if it exceeds *m*, and if it is shorter than *m*, it is zero-padded.

$L_{r}\in R^{m\times 20}$ , $H_{r}\in R^{m\times 20}$, and $A_{r}\in R^{m\times 20}$ represent the matrices of antibody heavy chain, antibody light chain, and antigen, respectively.

### 2.8 Matrix decomposition

We flattened the feature matrices of all sequences into a one-dimensional space and employed a matrix decomposition approach to integrate the antigen and antibody features, thereby establishing a connection between antigen and antibody sequences (Wang *et al.* 2023).

(7) $$F_{l}=L_{r}^{T}W_{l}+A_{r}W_{l},F_{h}=H_{r}^{T}W_{h}+A_{r}W_{h}$$

where $F_{l},F_{h}$ denotes the feature matrices of the light and heavy chains connected to the antigen, and $W_{l}$, $W_{h}$ denotes learnable parameter matrices.

### 2.9 MLP for residue feature

The feature matrices of light chains and heavy chains connected to the antigen were spliced and fed into a multi-layer perceptron model (MLP) to capture the interactions in complexes.

(8) $$\hat{y_{r}}=\text{MLP}\left(\left[F_{l},F_{h}\right]\right)$$

where ${\hat{y}}_{r}\in R$. MLP is a 2-layered MLP with ReLU as nonlinear activation layers.

### 2.10 Training and optimization

Sequence features and residue features were processed in parallel, and the outputs of CNN and MLP were weighted and averaged to obtain the final prediction:

(9) $$\hat{y}=\omega_{1}\hat{y_{s}}+\omega_{2}\hat{y_{r}},\omega_{2}=1-\omega_{1}$$

where $\omega_{1}\in \left(0,1\right)$ and $\omega_{2}\in \left(0,1\right)$ are hyperparameters that correspond to the weights of the outputs of CNN and MLP, respectively.

### 2.11 Experimental setting

The models were implemented in PyTorch and trained on NVIDIA GeForce RTX 4090 GPUs. Hyperparameter optimization was performed through 20 rounds of ephemeral training using the SAM optimizer with SGD momentum. The initial learning rate was set to 0.0001 and scheduled to decay during training. L1 regularization of 0.0001 was applied to mitigate overfitting, and L1 loss was used as the objective function. On the test set, the average time to process one antibody–antigen sample was 20.6 ms, while unobserved antigen–antibody pairs could be predicted every 15.6 ms on average.

In the “Holdout” experiment, we randomly divided the original set of samples into two parts, the training set and the validation set with a ratio of $9:1$.

In the standard “ten-fold cross-validation” experiment (Albu *et al.* 2023, Nikam *et al.* 2023), the dataset was first shuffled and divided into ten equal-sized subsets, called folds. The model is evaluated ten times, each time using a different fold as the testing set and the remaining nine folds as the training set. The overall performance is calculated by averaging the scores of the ten evaluations. For each evaluation, the samples in the testing set are not included in the corresponding training data. Importantly, the model is trained from scratch in each evaluation to ensure no overlap between training and test sets. Besides, each round of evaluation is conducted independently, ensuring that there is no data leakage from one evaluation to another. Supplementary Figure S1 illustrates how the testing set was distributed among the training sets. We can see that the testing set is independently and identically distributed from the training set.

## 3 Results

### 3.1 Performance on the natural dataset

**Figure 2.**
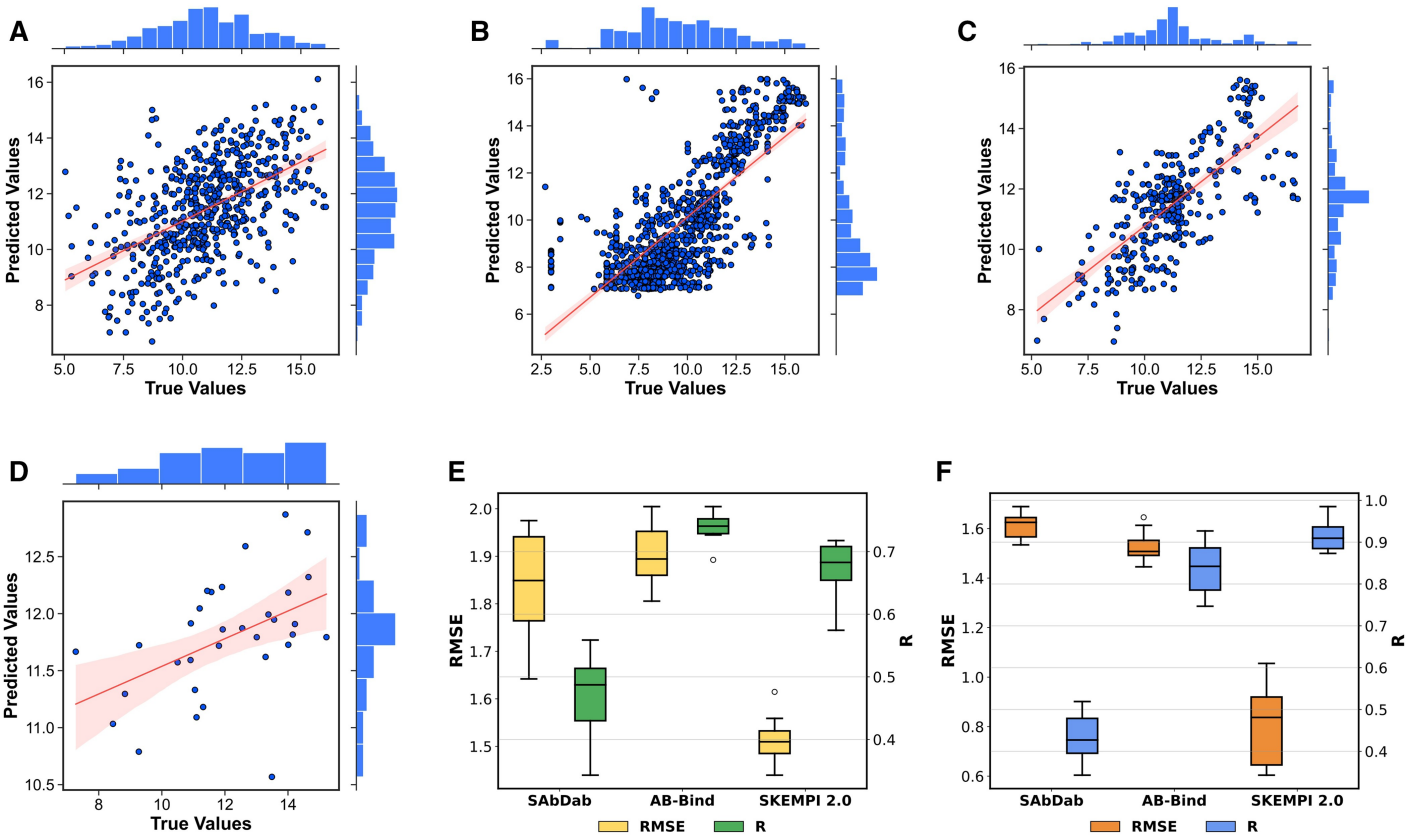
Performance of MVSF-AB on different datasets. A–D represent the scatter plot of true △G values versus predicted △G values obtained from 10-fold cross-validation on the SAbDab, AB-Bind, SKEMPI 2.0, and expanded benchmark datasets, respectively. E shows the RMSE and Pearson correlation coefficient (*R*) obtained from cross-validation experiments on three datasets. F shows the RMSE and *R* values obtained from the holdout experiments on three datasets.

Figure 2A depicts the scatter plot resulting from cross-validation on the natural dataset SAbDab. It is evident that there is a discernible linear relationship between the true and predicted affinity values (△G), indicating a positive correlation. However, this relationship exhibits a systematic bias. Specifically, when the true values are higher, the predicted values tend to slightly underestimate them. Conversely, when the true values are lower, the predicted values are inclined to slightly overestimate them.

Figure 2E presents the root mean square error (RMSE) and Pearson correlation coefficient (*R*) of cross-validation experiments on SAbDab dataset. MVSF-AB achieves an RMSE of 1.839 kcal/mol and a Pearson correlation coefficient of 0.491.

Figure 2F showcases the RMSE and *R* values of holdout experiments on SAbDab. Notably, the results are generally superior compared to those of cross-validation.

To evaluate the generalization ability in predicting the affinity of unknown natural antibodies, we followed the methodology outlined in (Yang *et al.* 2023) and assessed the effectiveness of our trained model on the expanded benchmark (Guest *et al.* 2021), which differs from the training dataset. Figure 2D presents the performance on the expanded benchmark datasets, with an RMSE of 1.447 kcal/mol and a Pearson correlation coefficient (R) of 0.467. These results showcase that MVSF-AB is superior in predicting the affinity of unknown natural antibodies and has remarkable generalization ability.

### 3.2 Adaptation on mutated datasets

Given that all antibodies in the SAbDab dataset are naturally occurring, it is necessary to validate whether our method is effective for mutant antibody data. In contrast to predicting the impact of point mutations on affinity, predicting the affinity of unknown natural antibodies can be regarded as a cold-start problem, which is more challenging.

We trained the model separately on the AB-Bind and SKEMPI 2.0 datasets and conducted 10-fold cross-validation. The results are shown in Fig. 2B and C, indicating a positive correlation. On the AB-Bind dataset, depicted in Fig. 2E, our model achieves an RMSE of 1.905 kcal/mol and a Pearson correlation coefficient of 0.739. Similarly, on the SKEMPI 2.0 dataset, our model yields an RMSE of 1.513 kcal/mol and a Pearson correlation coefficient of 0.671. These results demonstrate the effectiveness of our method when predicting the affinity of mutant antibodies. Figure 2F presents the results of holdout experiments, which outperform those of cross-validation experiments. This discrepancy arises from the randomness inherent in cross-validation, making it impossible to guarantee that each mutant in the test set has an adequate number of other mutants in the training set from which the model can learn.

Interestingly, our model exhibits superior performance on mutant antibodies than natural ones. This might be attributed to the greater sequence homology within the mutant dataset, resulting in a more data-dense and informative training environment (Ying 2019).

**Figure 3.**
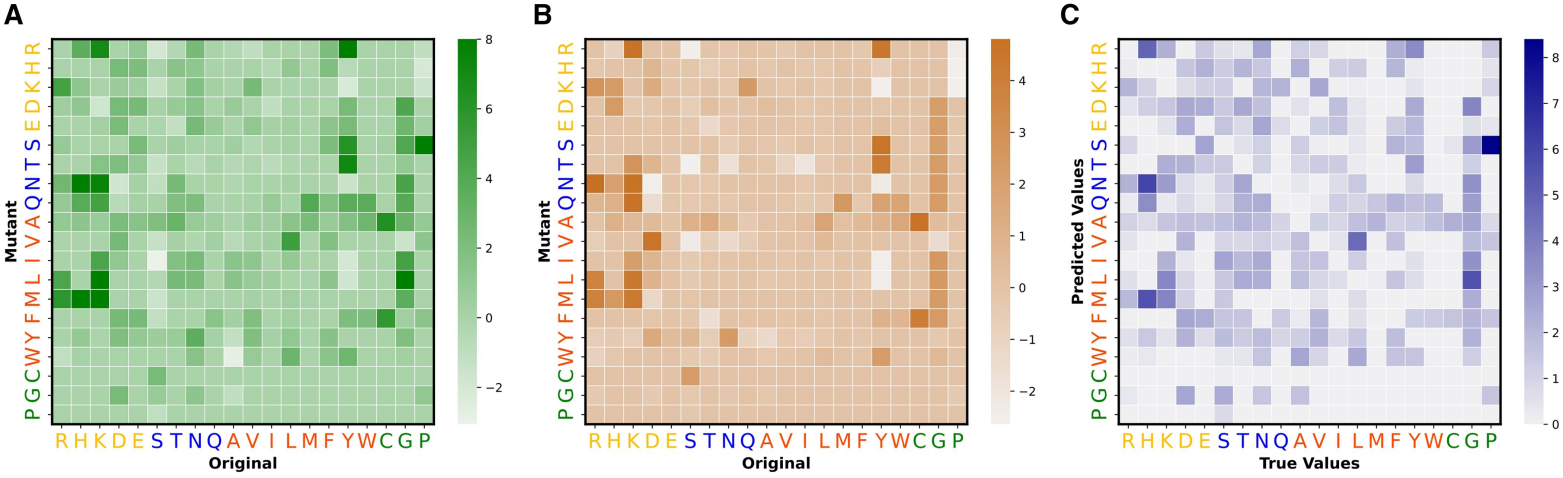
Impact of different amino acid mutations. A illustrates the true changes in the affinity induced by various mutations. B presents the predicted changes in the affinity resulting from various mutations. The *x*-axis labels the amino acid type of the original, while the *y*-axis labels the amino acid type of the mutant. The 20 classes of amino acids are visually distinguished by using fonts in different colors. The capital letters “R, H, K, D, E”, “S, T, N, Q”, “A, V, I, L, M, F, Y, W”, “C, G, P” are charged, polar, hydrophobic, and special-case amino acids, respectively. C presents errors between the predicted affinity and the true affinity for different mutations.

In addition, we analyzed the impact that different amino acid mutations have on affinity prediction. Figure 3B demonstrates the changes in predicted affinity resulting from mutations, quantified as △△G (kcal/mol). The predicted affinity is consistent with the true affinity presented in Fig. 3A. For instance, mutations in polar and hydrophobic amino acids result in substantial changes in affinity. Figure 3C presents errors between the predicted affinity and true affinity for different mutations. The figure reveals that mutations from amino acids S to P, and from amino acids R, N, and M to H, are more likely to result in relatively large prediction errors.

### 3.3 Comparison with existing works

Since protein-protein affinity prediction methods can be directly applied to antibody–antigen affinity prediction, we performed comparative experiments with existing four sequence-based models for predicting protein-protein affinity, including PPA-Pred2 (Yugandhar and Gromiha 2014), ISLAND (Abbasi *et al.* 2020), PIPR (Chen *et al.* 2019), and ProtALBERT (Unsal *et al.* 2022), which are recently proposed popular baselines, as depicted in Table 2. We also made scatter plots of the predicted values of the different methods in Supplementary Fig. S3.

**Table 2.** Results of MVSF-AB and baselines for antibody–antigen binding affinity prediction on different datasets.

		Holdout			Cross-validation		
Methods	Benchmark	SAbDab	AB-Bind	SKEMPI 2.0	SAbDab	AB-Bind	SKEMPI 2.0
PPA-Pred2 (Yugandhar and Gromiha 2014)	3.020 (−0.075)	—	—	—	—	—	—
ISLAND (Abbasi *et al.* 2020)	2.077 (0.212)	2.244 (0.116)	20.400 (−0.130)	2.188 (0.477)	2.076 (0.123)	19.499 (0.053)	1.889 (0.357)
PIPR (Chen *et al.* 2019)	1.679 (0.164)	1.963 (0.389)	1.583 (0.815)	0.998 (0.914)	2.070 (0.452)	1.927 (**0.761**)	1.662 (0.563)
ProtALBERT (Unsal *et al.* 2022)	1.930 (0.202)	1.944 (0.383)	1.560 (0.813)	1.244 (0.923)	2.338 (0.017)	3.556 (−0.210)	2.312 (0.023)
CSM-AB (Myung *et al.* 2022)	2.650 (0.364)	—	—	—	—	—	—
MVSF-AB	**1.447 (0.467)**	**1.608 (0.431)**	**1.526 (0.838)**	**0.808 (0.933)**	**1.839 (0.491)**	**1.905** (0.739)	**1.513 (0.671)**

The performance of antibody–antigen affinity prediction using RMSE and Pearson correlation coefficient (in parentheses). The best results are highlighted in bold. Note that PPA-Pred2 and CSM-AB only provide web servers and interfaces for testing, lacking the ability to be trained on the antibody dataset. Therefore, we only evaluate these two methods on the benchmark.

PPA-Pred2 (Yugandhar and Gromiha 2014) exhibits poor performance on the benchmark dataset, as evidenced by negative Pearson correlation coefficients. Since the original code was not provided, we are unable to evaluate its performance on the antibody dataset. The performance of ISLAND (Abbasi *et al.* 2020) when trained on these datasets was significantly below expectations. As depicted in Supplementary Fig. S3, the predictions converge to a common value, which is not correlated with the true values, which may be due to the model not capturing sufficient variability, and the features used for training cannot adequately explain the target variable. Additionally, upon reproducing the code, we found that the “propy” library used by ISLAND is overly restrictive in terms of feature extraction and is unsuitable for datasets with variable sequence lengths. PIPR (Chen *et al.* 2019) is a pioneering model for predicting protein affinity using neural networks, and its performance is stable across different datasets. There is a positive correlation relationship between the true and predicted values in PIPR and MVSF-AB. ProtALBERT (Unsal *et al.* 2022) is considered to be the most effective protein pretraining model. However, due to its inability to handle small data diversity and cold starts problems, it performed poorly in cross-validation experiments, which may stem from the difference in the distribution of the pretrained model’s training data compared to the antibody dataset we used.

Overall, our proposed method gets superior results on different datasets. The prediction on the mutant dataset is better than that of the natural dataset, and the results of the holdout experiments are better than that of the cross-validation experiments, especially for the mutant dataset.

In addition, we compare our scheme with CSM-AB (Myung *et al.* 2022), which predicts antibody–antigen affinity from structure information. The results in Table 2 show that MVSF-AB surpasses CSM-AB even though MVSF-AB only uses the sequence information. Due to the unavailability of the source code for CSM-AB to train on the antibody dataset, we only evaluate the performance of CSM-AB on the natural dataset SAbDab. There were 100 samples in the SAbDab dataset for which predictive values could not be obtained due to their inability to be recognized as antibodies by the CSM-AB, so we evaluated the performance of the remaining over 400 samples. CSM-AB achieved a RMSE of 1.80 and a Pearson correlation coefficient of 0.56, as detailed in Supplementary Fig. S4.

Note that the absence of information regarding the interfacial amino acids of the mutant antigen–antibody complex hinders the assessment of CSM-AB’s performance in a mutation scenario. This limitation underscores the limitations associated with the utilization of structure-based prediction methodologies.

### 3.4 Ablation study

#### 3.4.1 Multi-view versus single-view sequence feature

**Figure 4.**
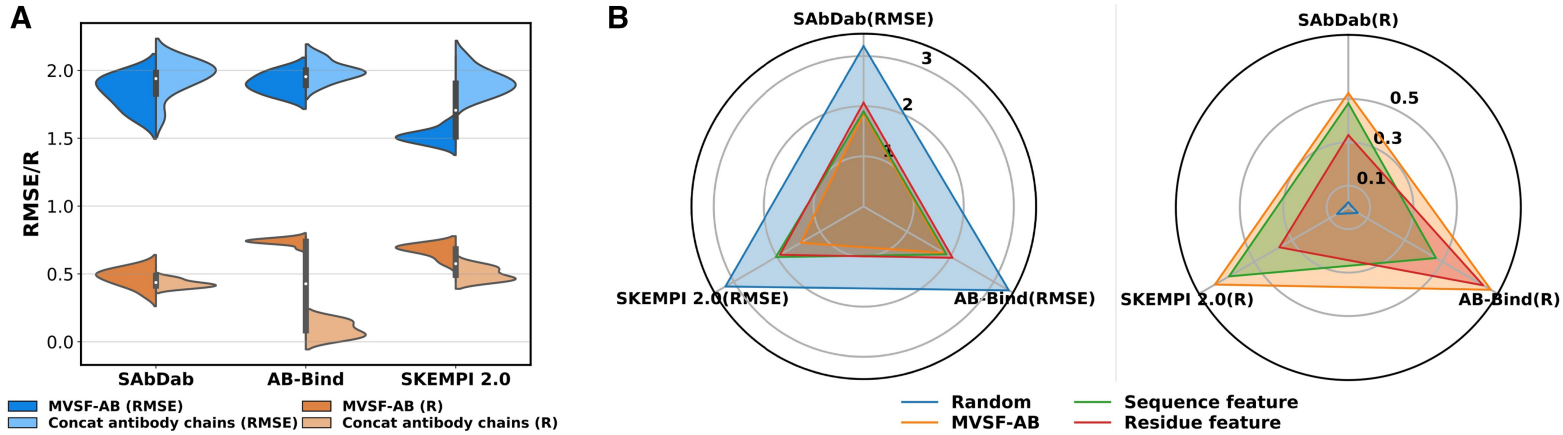
Performance of MVSF-AB under different settings. (A) Performance in which the light and heavy chains of the antibody are jointly inputted into the pretrained model. (B) Performance using single-view features.

The CNN is employed to capture the sequence semantic feature between antibody and antigen sequences during the binding process. The results presented in Fig. 4B indicate a moderate reduction in the observed effects when exclusively relying on sequence features, particularly when evaluated on the AB-Bind test set. This suggests that the combination of the CNN architecture and pretraining exhibits superior performance in feature extraction, surpassing existing methods in functional prediction.

The MLP is utilized to comprehend the contributions of amino acids with distinct physicochemical properties in the binding process (Pinkus 1999). The results depicted in Fig. 4B demonstrate a decline in effectiveness when trained exclusively on the residue features derived from AAindex. Specifically, the variant model that used only residue features performs less effectively compared to the one utilizing solely sequence features on the SAbDab and SKEMPI 2.0 test sets, while exhibiting the opposite trend on the AB-Bind test set.

In summary, our customized model demonstrates improved predictions for each single-view feature, but the integration of multi-view features could yield superior and stable performance, indicating that the model could learn comprehensive knowledge about antigen–antibody correlations from multi-view sequence features to substantially improve prediction accuracy.

#### 3.4.2 Feature fusion

For networks with multiple inputs, the design of feature fusion plays a critical role and significantly influences the feature learning process in the subsequent convolutional layer. In this study, we attempted to linearly concatenate the features of the antibody light and heavy chains after separately embedding the corresponding chains of the antigen and antibody. These concatenated features were then fused with the antigen features and subjected to the same convolutional operation. The feature fusion process is represented as follows:

(10) $$D=\left[\left|L_{s}⊖A_{s}\right|,\left|H_{s}⊖A_{s}\right|\right],$$

 

(11) $$\begin{array}{c} P=\left[L_{s}⊙A_{s},H_{s}⊙A_{s}\right],C=\left[D,P\right] \end{array}$$

where $C\in R^{2\times 1536\times 768}$ denotes the fused feature tensor. However, the prediction results turn out to be worse than random guessing. We subsequently proceeded to input the light and heavy chains of the antibody as a unified sequence into the embedding layer, followed by fusion with the antigenic features and subsequent convolution.

(12) $$Ab_{s}=\text{Embed}\left(\left[L,H\right]\right),{\text{Ag}}_{s}\;=\;\text{Embed}\left(A\right)$$

where $Ab_{s}$ and $Ag_{s}$ are 768-dimensional vectors. The results in Fig. 4A show a slight improvement in this variant model, yet it remains inferior to the MVSF-AB on all test sets. This suggests that our convolution strategy is designed properly to align with the output of the pretrained model, and a single channel can only represent the features between the global embedding features of a pair of sequences. In scenarios involving multiple pairs of interacting sequences, treating the fused features of each sequence pair as separate channels can enhance the accuracy, as demonstrated by MVSF-AB.

## 4 Discussion

In the realm of predicting general protein-protein affinity, significant efforts have been devoted to developing predictive models. These models can be applied to specific proteins such as antibodies. However, our experimental findings reveal that these methods for general protein, when applied to antibodies, may not yield the expected results. This underscores the necessity for tailoring specialized models catering to distinct protein types to enhance prediction accuracy.

We further validate the effectiveness of fusing features from multiple perspectives rather than using individual features, thereby enhancing the comprehension of antigen–antibody binding affinity. While the approach of extracting features via pretrained language models is gaining traction in amino acid sequence research, it is vital not to disregard the valuable insights provided by prior knowledge (Wang *et al.* 2021, Zhang *et al.* 2022), such as the inherent properties of amino acids. The fusion of multi-view features is effective not only in antibody affinity prediction but also in other sequence analyses. Consequently, there is ongoing potential to explore additional sequence features from diverse viewpoints.

Given the limited antigen–antibody datasets that provide affinity tags, it is essential to effectively extract the feature of antibody sequences by leveraging pretrained language models “ProteinBERT.” In the future, we can fine-tune the pretrained model using specific antibody and antigen sequences. This approach will facilitate the development of a tailored pretrained model capable of accurately processing the antigen–antibody sequence information. Concurrently, research efforts have been directed toward predicting active sites (Zeng *et al.* 2020, Zhao *et al.* 2020). In the future, leveraging data about active sites and significant contacts may be better than only using sequence data. This approach would not only reduce computational costs but also mitigate the impact of noise in the sequence.

## 5 Conclusion

In this study, we propose a novel framework designed explicitly for predicting antibody–antigen affinity, which not only excels in predicting the affinity of natural antibodies but also forecasts the affinity of antibody mutant strains. MVSF-AB extracts knowledge from multi-view sequence features, which maximizes the utilization of global and local features from antibody and antigen sequences and the properties of residues. By incorporating these features, we achieved effective predictions of their affinities, outperforming existing works. This provides a fresh perspective on sequence analysis studies of protein complexes.

## Supplementary Material

btae579_Supplementary_Data

## Data Availability

The SAbDab dataset is available at https://opig.stats.ox.ac.uk/webapps/sabdab-sabpred/sabdab; the AB-Bind dataset is available at https://onlinelibrary.wiley.com/doi/10.1002/pro.2829; the SKEMPI 2.0 dataset is available at https://life.bsc.es/pid/skempi2/; and the AAindex dataset is available at https://www.genome.jp/aaindex/.
