# Supplementary material for: MVSF-AB: accurate antibody–antigen binding affinity prediction via multi-view sequence feature learning
Source: Bioinformatics. 2024 Oct 3;41(5):btae579. doi: 10.1093/bioinformatics/btae579 (PMC12089643; doi:10.1093/bioinformatics/btae579)
Supplement: btae579_Supplementary_Data [file btae579_supplementary_data.pdf]

Supplementary material

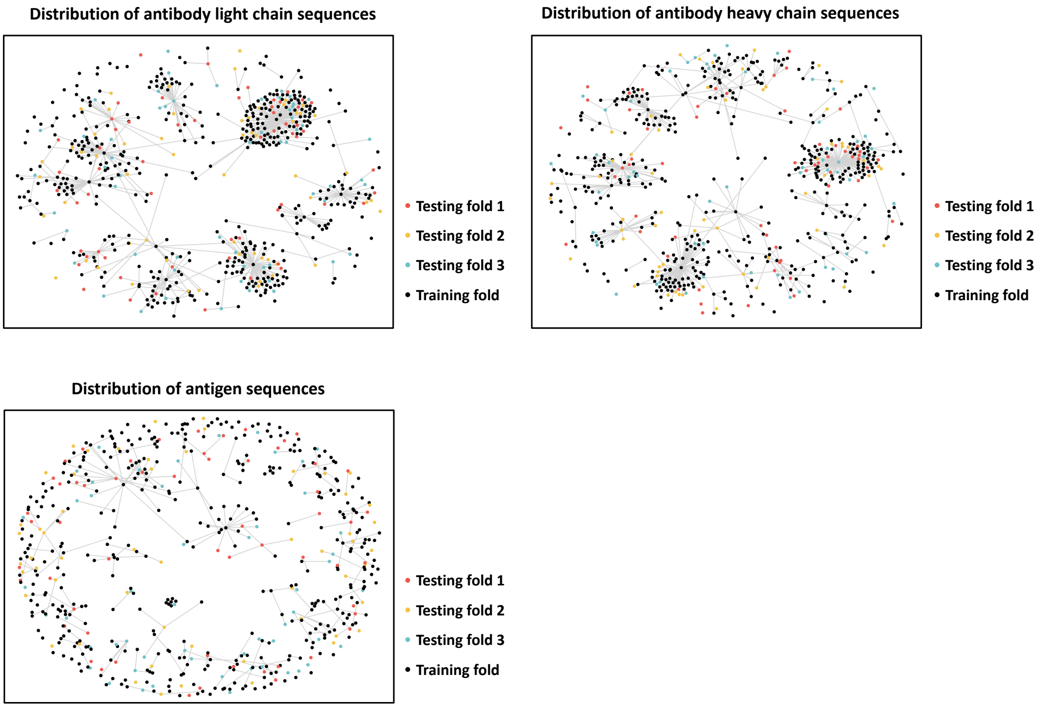

Figure S1. The distribution and sequence similarity between the testing and training sets in the ten-fold cross-validation. Similarity for antibody/antigen sequences was computed using CD-HIT (Cluster Database at High Identity with Tolerance), a widely used bioinformatics tool for clustering and comparing protein or nucleotide sequences. Each node represents an individual sequence, and nodes are connected by a line if their similarity is 80% or greater. A longer line indicates lower similarity. The proximity of two dots without a connecting line does not imply similarity between the corresponding sequences.

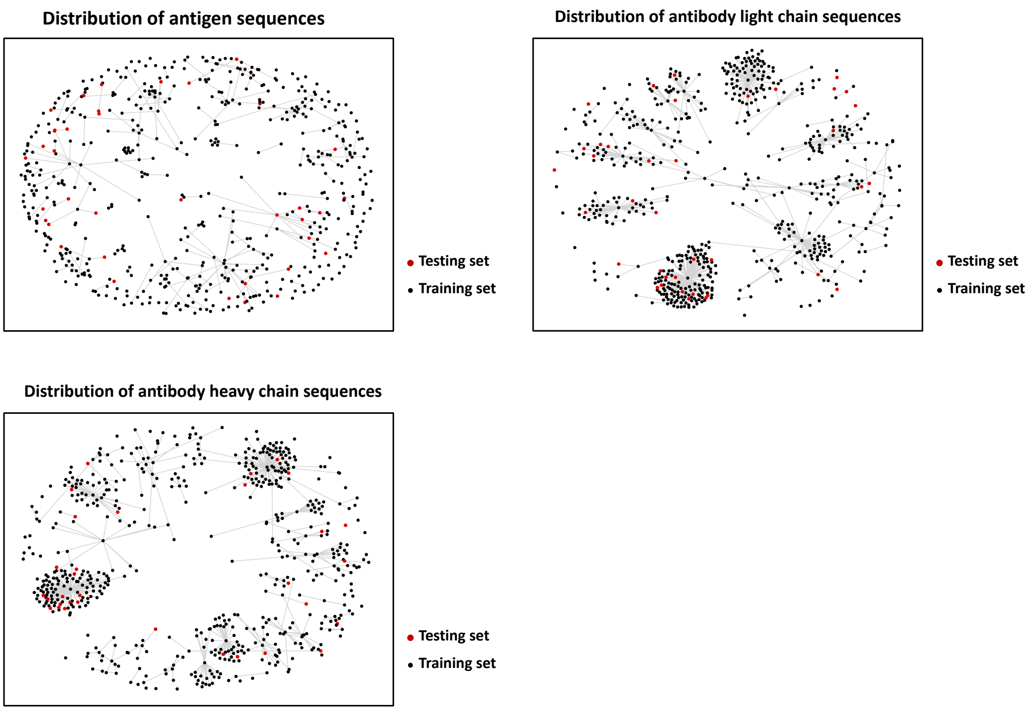

Figure S2. The distribution and sequence similarity between the benchmark as testing set and SAbDab as training set.

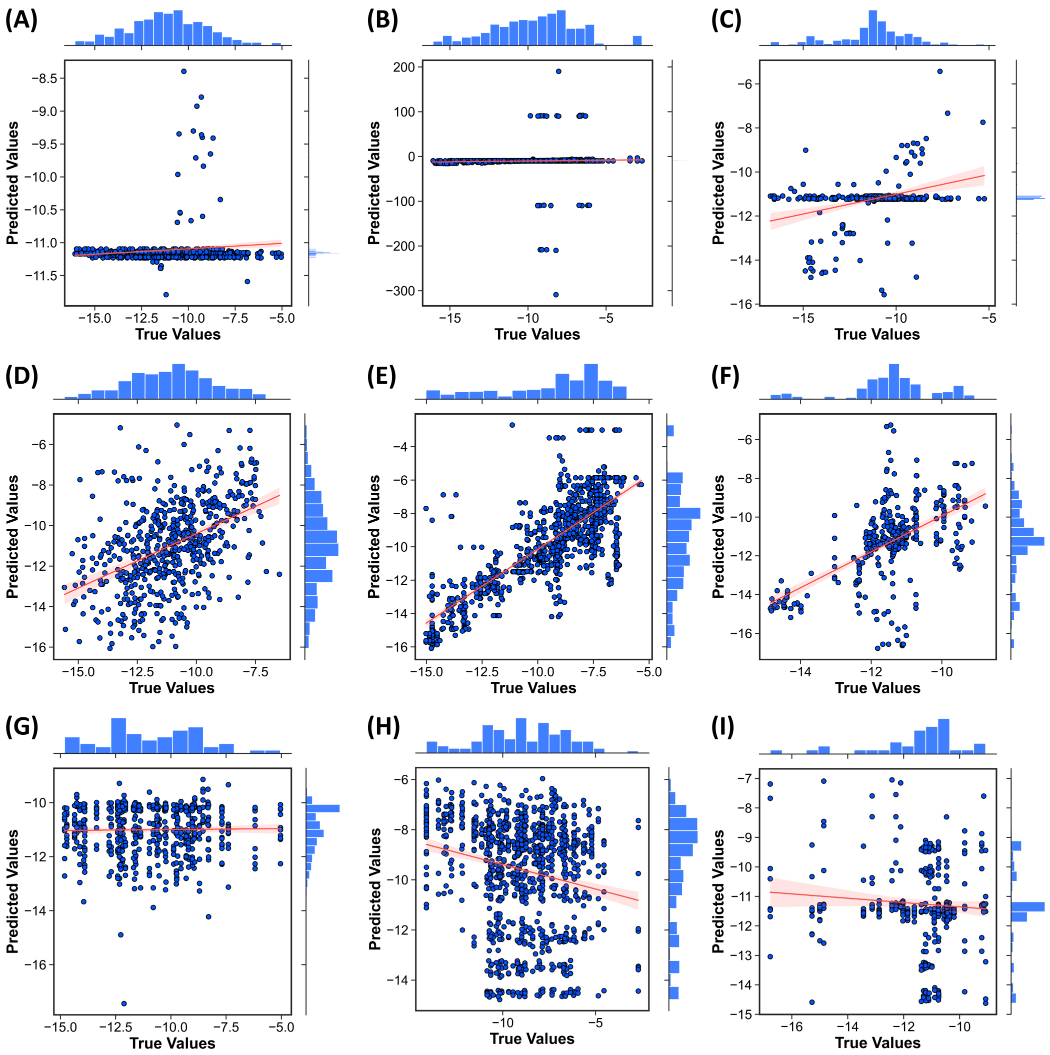

Figure S3. Scatter plot of true ΔG values *vs* predicted ΔG values for ISLAND, PIPR and ProtALBERT on SAbDab, AB-Bind and SKEMPI 2.0 datasets. Figures S3.A, S3.B and S3.C represent the scatter plot of ISLAND on SAbDab, AB-Bind, and SKEMPI 2.0 datasets, respectively. It was observed that the predictions of this method converge to a common value, which is not correlated with the true values. Figures S3.D, S3.E and S3.F represent the scatter plot of PIPR on SAbDab, AB-Bind, and SKEMPI 2.0 datasets, respectively. There is a positive correlation relationship between the true and predicted values. Figures S3.G, S3.H and S3.I represent the scatter plot of ProtALBERT on SAbDab, AB-Bind, and SKEMPI 2.0 datasets, respectively. There is no correlation or negative correlation relationship between the true and predicted values, which may stem from the difference in the distribution of pre-trained model's training data compared to the antibody dataset we used.

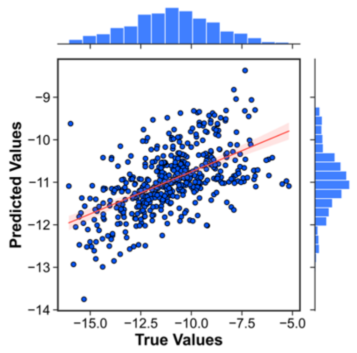

Figure S4. Performance of CSM-AB on the SAbDab dataset.

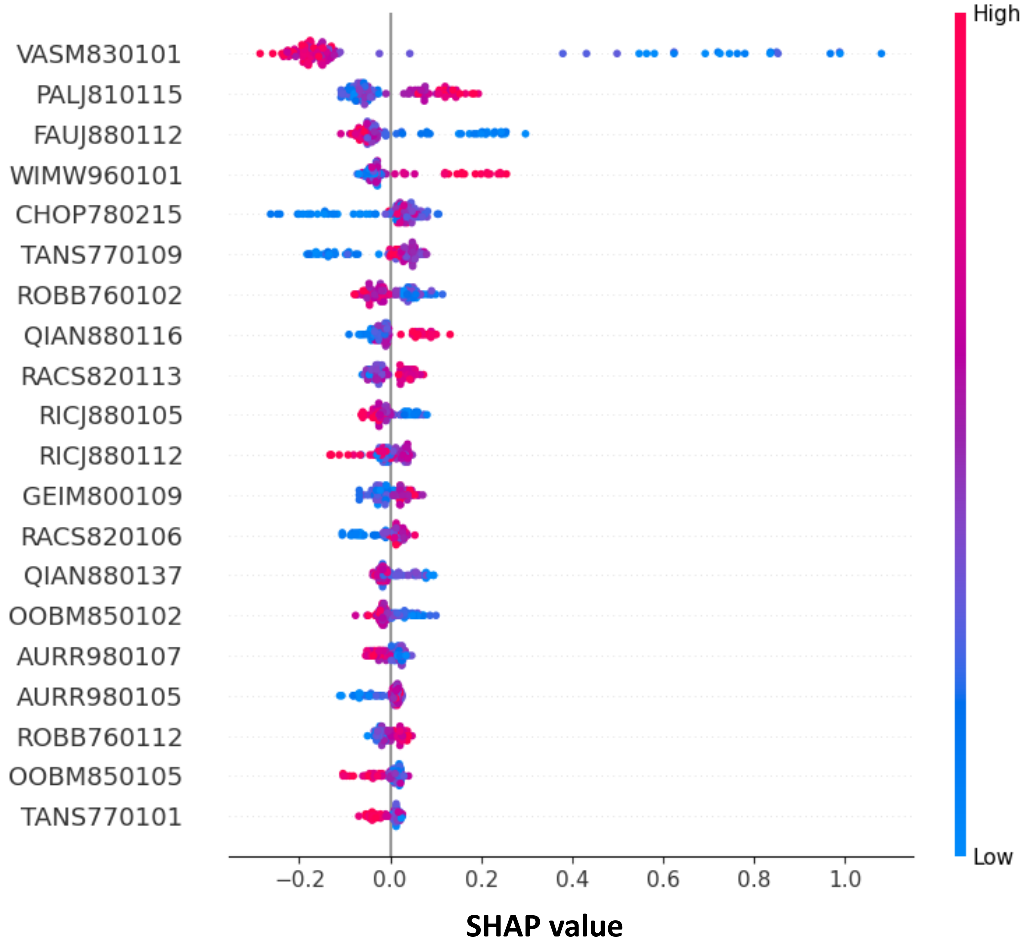

Figure. S5. Top 20 AAindex features ranked by their importance in predicting antigen-antibody affinity. Most of these features are predominantly associated with conformational properties or negative chargeability.
